# Evaluation of the Antibacterial and Antifungal Efficacy of Chitosan Nanoparticles in Irreversible Hydrocolloid Impression Materials: A Cross-Sectional Study

**DOI:** 10.7759/cureus.78414

**Published:** 2025-02-03

**Authors:** Bharat Kumawat, Sandeep Kumar, Rajnish Aggarwal, Gita Rani, Shabnam Choudhary, Jaspinder Kaur, Ashutosh R Singh

**Affiliations:** 1 Department of Prosthodontics, Surendera Dental College and Research Institute, Sri Ganganagar, IND; 2 Department of Prosthodontics, ITS Dental College, Ghaziabad, IND

**Keywords:** alginate, antibacterial, antifungal, chitosan, disinfectant, nanoparticles

## Abstract

Introduction: Irreversible hydrocolloid (IHC) impression materials are widely used in dentistry for diagnostic impressions; however, they pose a risk of cross-contamination. Current cleaning methods such as rinsing with water may not be sufficient for effective disinfection. Incorporation of antimicrobial agents into IHC materials can reduce this risk. Chitosan, a biopolymer with proven antibacterial and antifungal properties, has potential as a self-disinfecting agent for IHC materials. This study aimed to evaluate the antibacterial and antifungal efficacy of chitosan nanoparticles (CHN) incorporated into IHC (alginate) impression materials.

Materials and methods: This cross-sectional study was conducted in the Department of Prosthodontics, at the Surendera Dental College and Research Institute, Sri Ganganagar, Rajasthan, India, with 20 dentulous patients. A 1% CHN was incorporated into the IHC impression material to serve as Group 1, while conventional alginate mixed with distilled water with a one-week interval between each impression served as Group 2. Impressions were made on patients' maxillary arches, with bacterial and fungal samples collected at 0- and 10-minute intervals after rinsing the impressions. The samples were cultured on nutrient agar and Sabouraud dextrose agar for bacteria and fungi, respectively. Colony counts were assessed and statistically analyzed.

Results: The study found a significant reduction in both bacterial and fungal counts in impressions made with Group 1 compared with Group 2 at both 0- and 10-minute intervals (p<0.001). At 10 minutes, bacterial counts decreased significantly in Group 1 (from 75.50±34.56 to 26.00±16.03), and fungal counts were also reduced (from 2.70±1.22 to 0.55±0.69). The antibacterial and antifungal efficacy was increased at the 10-minute interval, compared to the 0-minute interval.

Conclusion: Incorporating 1% CHN into the IHC impression material significantly enhanced its antibacterial and antifungal properties, making it an effective self-disinfecting agent. This modification reduces the need for additional disinfection steps and offers a practical solution for improving cross-infection control in dental practice. CHN's biocompatibility and biodegradability further support its potential as an eco-friendly and sustainable alternative for dental materials.

## Introduction

Dentistry employs a diverse array of irreversible hydrocolloid (IHC) impression materials for accurate, conclusive, and diagnostic impression methodologies [1]. During the formation of diagnostic or definitive impressions, the impression material interacts with the patient's blood, saliva, biofilm, and plaque [2]. These interactions augment the potential for cross-contamination and infection as they facilitate the transmission of pathogens from the patient to the dentist, their personnel, and/or their laboratory technicians [3].

It has been substantiated that impressions derived from immunohistochemistry could be deemed adequate contingent upon rinsing with potable water. The act of rinsing under running tap water for 10-15 seconds has been shown to eliminate approximately 40% of the bacterial presence and can diminish the bacterial load by as much as 90% [4]. Nevertheless, alternative scholars contend that cleaning alone does not confer adequate protection [5].

To mitigate the potential for infection, it has been recommended that antiseptic solutions or disinfection protocols be implemented immediately following the removal of the impression from the oral cavity. The creation of irreversible, self-sterilizing hydrocolloid impression materials (alginate integrated with povidone-iodine powder and chlorhexidine) was driven by the challenges associated with the external sterilization of these substances [6]. Given that disinfectants are uniformly distributed throughout the material, self-disinfecting IHC materials possess the significant advantage of being sterilized both internally and externally [7].

The deacetylation of chitin yields a naturally occurring, non-toxic biopolymer, referred to as chitosan. Chitin is predominantly found in the cell walls of fungi and in the exoskeletons of arthropods, such as lobsters and shrimp. This versatile hydrophilic polysaccharide is characterized by its low toxicity to mammalian cells, extensive efficacy, rapid lethality, and potent antibacterial and antiviral properties [8]. Considering its numerous advantages, exceptional adaptability, remarkable biodegradability, biocompatibility, antibacterial properties, and non-toxic nature, chitosan has been widely recognized as one of the most promising biomaterials in the 21st century. In the field of dentistry, chitosan is utilized as a dental adhesive, serves as a coating for implants, and is incorporated into mouthwashes and toothpaste to reduce plaque accumulation [9].

Although numerous substances have been employed in the development of self-disinfecting IHC impression materials, no definitive standard or universally accepted material currently exists. The principal aim of this investigation was to evaluate the antibacterial and antifungal efficacy of chitosan nanoparticles (CHN) incorporated into IHC (alginate) impression materials. The secondary objective included determining whether CHN could function effectively as an antibacterial and antifungal agent within alginate impression materials across various time intervals.

## Materials and methods

This cross-sectional study was conducted in the Department of Prosthodontics, at the Surendera Dental College and Research Institute, Sri Ganganagar, Rajasthan, India, spanning the period from August 2023 to December 2023. Approval was secured from the institute's Institutional Ethics Committee (approval number: SDRI/IEC/22/34), and the study adhered to the principles outlined in the Declaration of Helsinki. Written informed consent was obtained from all participants involved in the study. All the clinical and laboratory procedures were performed by a single operator to mitigate variability.

The study was conducted on 20 dentulous patients who were reported in the Outpatient Department, including patients aged more than 18 years, of any sex, with at least 10 teeth in the maxillary arch, and with normal salivary flow, non-smokers, non-tobacco chewers, and non-alcoholics. Patients who were using antimicrobial mouthwashes in the last three months, who were using any antiseptic or antibiotics, with active periodontal or endodontic disease, and with a previous history of orthodontic treatment were excluded from the study. The sample was kept as 20 patients based on power analysis by the G*Power statistical software (Version 3.6.9, Heinrich-Heine-Universität Düsseldorf, Düsseldorf, Germany) at 80% power, 95% confidence interval, and 5% alpha error, based on a previous study [10].

**Figure 1:**
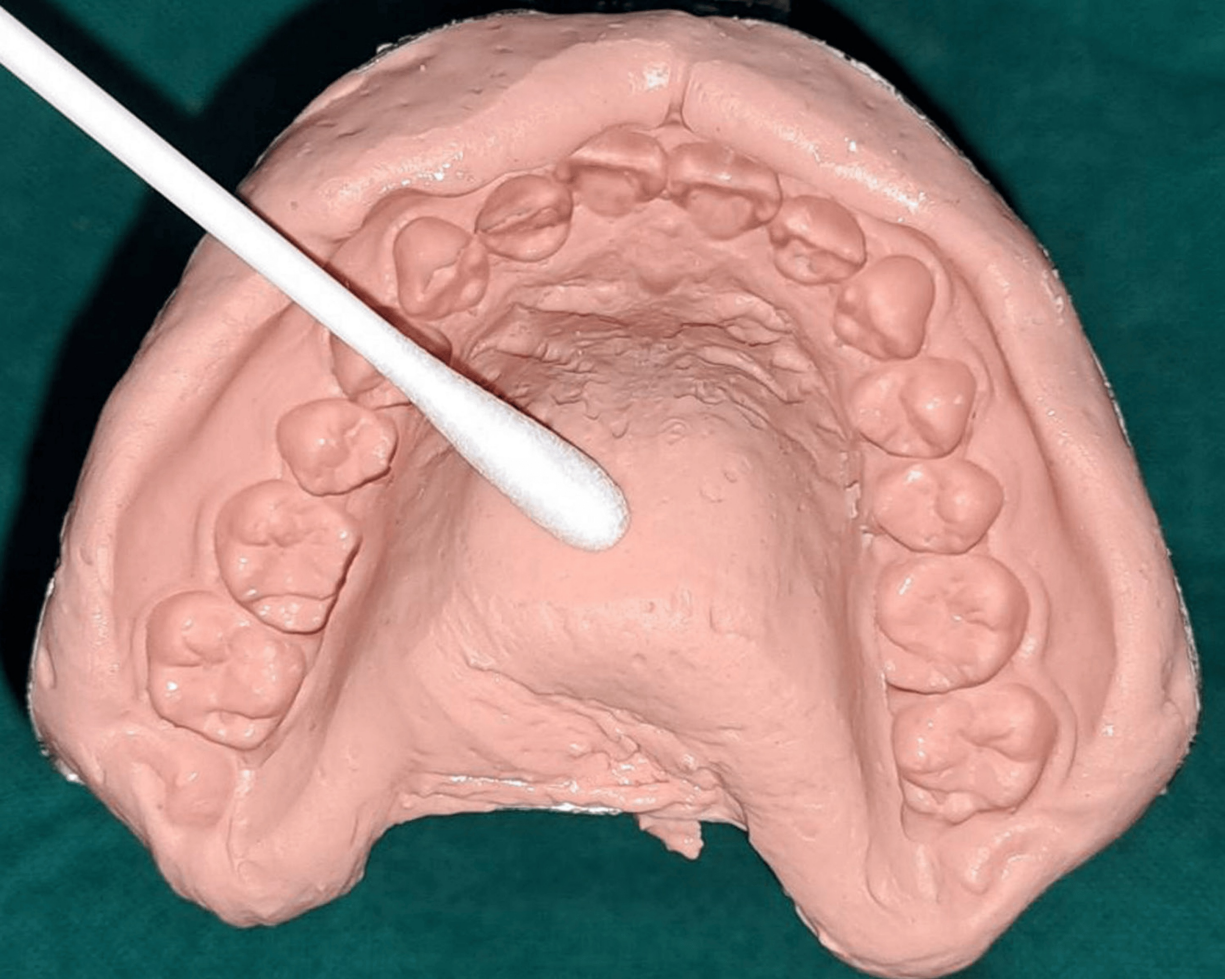
The samples were procured from the left, right, and mid-palatal regions with a swab. Source: The image is of an impression obtained from the patient in the study and used with permission.

CHN (Nanochemazone, Leduc, Alberta, Canada) with a purity of more than 99.9% and an average particle size of 80-100 nm was used in this study. To prepare 1% CHN, 1 g of CHN was added to 99 g of alginate (IHC) powder (CDH Fine Chemical, New Delhi, India) in a blender and mixed evenly. The mixture was stored in an airtight container for further use. Each participant had two maxillary impressions: one with 1% chitosan-impregnated alginate impression material (Group 1) and another with alginate mixed with distilled water in a conventional manner, according to the manufacturer's instructions (Group 2). After taking the first impression with either CHN or distilled water, the patient was recalled for a second impression after one week to allow the recovery of bacterial growth. To remove bacteria or any other contaminants from the impression, impressions were rinsed for 10-15 seconds in distilled water. After rinsing, sterile cotton swabs were used to acquire bacterial and fungal specimens. At intervals of 0 and 10 minutes, the samples were procured from the left, right, and mid-palatal regions (Figure 1).

**Figure 2:**
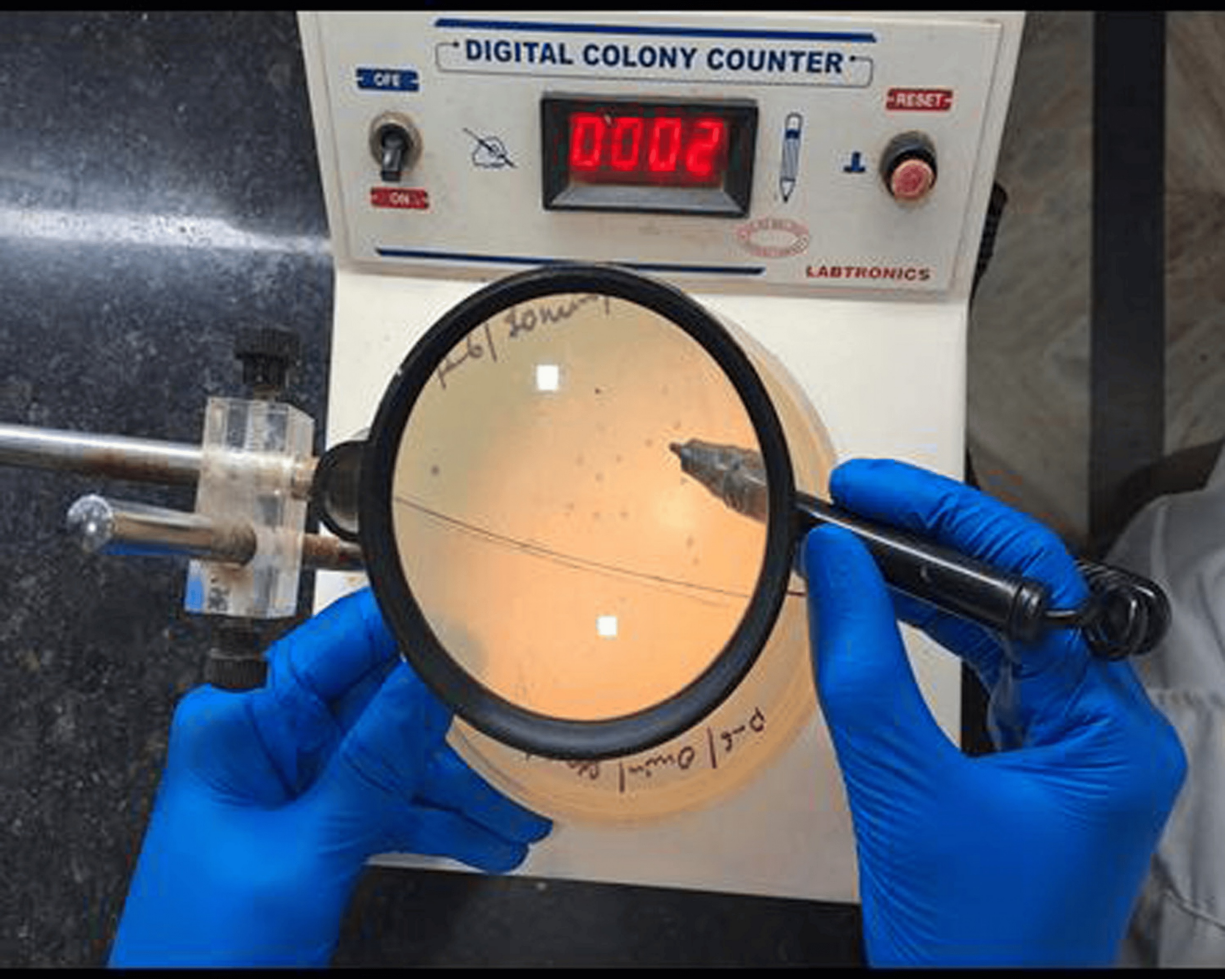
Digital colony counter (Labtronics, Panchkula, India). Source: The image is of an instrument used in our study.

To ensure the preservation of the integrity of the impression, the swabs were meticulously encased within a zip-lock bag and maintained at a temperature range of 2-8°C within a sample transport box until they were transferred to the laboratory for bacterial and fungal cultures. Bacterial swabs were processed using the sample dilution method. This method involved taking a test tube, filling it with 1 mL of sterile peptone water, and stirring a swab sample to create a stock solution. Seven test tubes were used, each containing 1 mL of sterile peptone water. 0.1 mL of the stock solution was now transferred to the first test tube. From the first test tube, a 0.1 mL solution was added to the second test tube, and so on, until the seventh tube. After the dilution procedure, 0.1 mL of the final dilution from the seventh test tube was taken through a pipette and transferred to petri dishes containing bacterial growth media (nutrient agar media) by the spread plate method. After the inoculation procedure, the bacterial petri plates were incubated for 24-48 hours at 37°C. The final colony count was calculated by multiplying the final number of colonies by the dilution factor. Fungal swabs were directly inoculated on petri plates containing fungal growth media (Sabouraud dextrose agar). Chloramphenicol was added to stop the growth of the bacterial colonies. After the inoculation procedure, fungal culture plates were incubated for seven days at 25°C in a microbial incubator. Colony-forming units for each culture were counted using a digital colony counter (Labtronics, Panchkula, India) (Figure 2).

Statistical analysis

The results were statistically analyzed using IBM SPSS Statistics for Windows, Version 19.0 (Released 2010; IBM Corp., Armonk, New York, United States). Means and standard deviations were computed for the descriptive statistics. The Shapiro-Wilk test was used to check the normality of the data before analysis, and the results indicated that the data were normally distributed. Thereafter, the bacterial and fungal counts between the two groups at various time periods were compared using independent t-tests. The intragroup comparison of changes in bacterial and fungal counts between 0 and 10 minutes in the two groups was performed using a paired t-test. For the current investigation, a p-value of less than 0.05 was established as the threshold of significance. A p-value of less than 0.001 was considered highly significant.

## Results

The mean age of the 20 patients was 35.56±5.13 years, with a sample comprising 12 (60%) males and eight (40%) females. There was a statistically significant decrease in bacterial count in impressions taken with CHN-incorporated alginate compared to conventional alginate impressions at both time intervals (0 and 10 minutes) (p<0.001). This showed that CHN significantly reduced bacterial counts and was effective as an antibacterial agent (Table 1).

**Table 1:** Intergroup comparison of mean bacterial colony-forming units at various time intervals in 20 patients. *p<0.001: highly statistically significant using the independent t-test Data is presented in the form of mean and standard deviation (SD).

Time interval	Groups	N	Mean	SD	t-value	p-value
Bacterial count at 0 minutes	Conventional alginate impression (Group 2)	20	681	249.08	10.77	<0.001*
	Chitosan-incorporated alginate impression (Group 1)	20	75.5	34.56		
Bacterial count at 10 minutes	Conventional alginate impression (Group 2)	20	331.5	164.87	8.25	<0.001*
	Chitosan-incorporated alginate impression (Group 1)	20	26	16.03		

Likewise, a highly statistically significant variation was observed in fungal count at both time intervals between the groups. Group 1 showed decreased fungal counts compared to Group 2 (p<0.001). This showed that CHN significantly reduced fungal counts and was effective as an antifungal agent (Table 2).

**Table 2:** Intergroup comparison of mean fungal colony-forming units at various time intervals in 20 patients. *p<0.001: highly statistically significant using the independent t-test Data is presented in the form of mean and standard deviation (SD).

Time interval	Groups	N	Mean	SD	t-value	p-value
Fungal count at 0 minutes	Conventional alginate impression (Group 2)	20	6.3	2.03	6.80	<0.001*
	Chitosan-incorporated alginate impression (Group 1)	20	2.7	1.22		
Fungal count at 10 minutes	Conventional alginate impression (Group 2)	20	2.4	1.27	5.72	<0.001*
	Chitosan-incorporated alginate impression (Group 1)	20	0.55	0.69		

Table 3 shows the comparison of changes in bacterial and fungal counts at various time periods in Group 2. The results of the paired t-test indicated that there was a highly statistically significant difference in bacterial counts at 0 and 10 minutes (p<0.001). There was a significant reduction in the mean bacterial count from 0 minutes (681.00±249.08) to 10 minutes (331.50±164.87). Likewise, a statistically significant variation was observed in fungal count at 0 and 10 minutes (p<0.001). There was a significant reduction in the mean fungal count from 0 minutes (6.30±2.03) to 10 minutes (2.40±1.27).

**Table 3:** Intragroup comparison of mean bacterial and fungal colony-forming units in conventional alginate impression (Group 2). *p<0.001: highly statistically significant using the paired t-test Data is presented in the form of mean and standard deviation (SD).

Time intervals	N	Mean	SD	t-value	p-value
Bacterial count at 0 minutes	20	681.00	249.08	5.23	<0.001*
Bacterial count at 10 minutes	20	331.50	164.87		
Fungal count at 0 minutes	20	6.30	2.03	7.23	<0.001*
Fungal count at 10 minutes	20	2.40	1.27		

Table 4 shows a comparison of changes in bacterial and fungal counts at various time periods in Group 1. The results of the paired t-test statistical analysis indicated that there was a highly statistically significant difference in bacterial count at 0 minutes and at 10 minutes (p<0.001). There was a significant reduction in the mean bacterial count from 0 minutes (75.50±34.56) to 10 minutes (26.00±16.03). Likewise, a statistically significant variation was seen in fungal count at 0 minutes and at 10 minutes (p<0.001). There was a significant reduction in the mean fungal count from 0 minutes (2.70±1.22) to 10 minutes (0.55±0.69).

**Table 4:** Intragroup comparison of mean bacterial and fungal colony-forming units in 1% chitosan-incorporated alginate impression (Group 1). *p<0.001: highly statistically significant using the paired t-test Data is presented in the form of mean and standard deviation (SD).

Time intervals	N	Mean	SD	t-value	p-value
Bacterial count at 0 minutes	20	75.50	34.56	5.81	<0.001*
Bacterial count at 10 minutes	20	26.00	16.03		
Fungal count at 0 minutes	20	2.70	1.22	6.86	<0.001*
Fungal count at 10 minutes	20	0.55	0.69		

## Discussion

The Centers for Disease Control and Prevention recommends the implementation of careful and thorough sanitation procedures for all items that come into contact with the oral cavity to eradicate the presence of blood and saliva. It is of utmost importance to ensure that the materials, impressions, and intraoral devices are properly cleaned and disinfected prior to their manipulation within the dental laboratory and their subsequent utilization within the patient's oral environment [11]. The implementation of cross-infection control protocols is of paramount importance for safeguarding patient safety. Disinfection of impressions constitutes an essential strategy that can significantly reduce the risk of infection transmission among dental clinics, laboratory personnel, patients, and dental support staff [12].

The antibacterial agent utilized as a disinfectant must satisfy two fundamental criteria: preservation of the surface details of the impression and maintenance of dimensional stability. In comparison to prior disinfection methodologies, it is posited that the implementation of a disinfectant-enhanced IHC impression material may yield superior efficiency and efficacy concerning both the impression and resultant cast. The impression material can achieve self-disinfection by integrating an antimicrobial agent, either through the amalgamation of the IHC impression material with a solution containing the antibacterial agent or by incorporating the agent in its powdered form into the IHC impression material [13]. Most previous studies used the first approach for self-disinfection of IHC impression materials [10,14,15]. The second method of incorporating the antibacterial agent in powdered form into the impression material is generally better because it allows for a more controlled and uniform distribution of the antimicrobial agent. It is also less likely to interfere with the physical or mechanical properties of the impression material because the powdered form can be blended seamlessly without introducing excess liquid. This method helps to maintain the integrity and performance of the impression material while achieving effective self-disinfection. Therefore, in the present study, powdered CHN was added to the IHC impression material.

Chitosan has been documented to exhibit properties that are non-toxic, antibacterial, biocompatible, and biodegradable. Its well-established characteristics include hemostatic, fungistatic, antibacterial, anticancer, anticholesteremic, and immunoadjuvant properties. In dentistry, chitosan serves as an effective antibacterial and antimicrobial agent. It has also been noted that spraying high-molecular-weight chitosan disinfectants does not alter the properties of polyvinylsulfide impression materials [16]. In this study, a concentration of 1% CHN was employed, according to the research conducted by Manikyamba et al. [10].

In the present study, bacterial and fungal colonies were assessed at 0- and 10-minute intervals to evaluate the rate of microbial mortality and the potential of chitosan, considering that between making the impression and pouring the cast, there could be a 0- to 10-minute delay during the transfer of the impression to the laboratory. The results of our study confirmed that 1% CHN was effective as an antibacterial and antifungal agent that can be used as a water substitute in IHC impression materials, which is in accordance with the studies of Bae et al. [17] and Manikyamba et al. [10]. However, both studies used chitosan in soluble form, and not CHN, as in our study. CHN demonstrates superior antibacterial efficacy compared to chitosan, attributable to the unique characteristics of the nanoparticles, presumably owing to the enhanced surface area and increased affinity with bacterial cells, which results in a quantum size effect [18].

Chitosan, characterized by its polycationic properties, contains NH3+ functional groups distributed along its polymeric backbone, which facilitate binding interactions with cross-linking agents, predominantly polyanions, such as tripolyphosphate ions. Conversely, alginate engages in cross-linking through its interactions with bivalent cations, such as calcium, thereby establishing a distinct mechanism compared to chitosan. The occurrence of this cross-linking phenomenon culminates in the generation of interlinked polymeric structures, which produce nanosized cavities capable of encapsulating pharmacological substances. This substantial cross-linking mechanism leads to the formation of stable nanoparticle frameworks that can enhance the structural stability of the alginate network and improve the mechanical properties of alginate, such as strength and elasticity, owing to the formation of these electrostatic bonds [19,20].

The predominant hypothesized antibacterial mechanism of chitosan involves its interaction with the negatively charged bacterial cell wall, which leads to cell disruption, thereby modifying membrane permeability, followed by its binding to DNA, resulting in the inhibition of DNA replication, ultimately leading to cell death. An alternative mechanism suggests that chitosan functions as a chelating agent, selectively binding to trace metal elements, which in turn promotes toxin production and hampers microbial proliferation [21]. CHN facilitates electrostatic interactions with the negatively charged membranes of fungal cells, resulting in augmented membrane permeability, compromised cellular wall integrity, and exfiltration of intracellular constituents, culminating in the death of fungal cells. Furthermore, chitosan can traverse the fungal cell wall and associate with intracellular entities such as DNA, thereby impeding replication and other essential biological processes [22].

The decreased antibacterial and antifungal activities at 0 minutes, which increased at 10 minutes, were observed in our study, which was in agreement with those reported by Manikyamba et al. [10]. The reason for this time-dependent effect may be due to the fact that alginate is rich in negatively charged carboxyl groups, while chitosan is positively charged. Initially, strong electrostatic interactions between alginate and chitosan may inhibit nanoparticle mobility, reducing antimicrobial activity. As the interaction equilibrates over time, CHN may detach or reposition to exert their antimicrobial effects. Moreover, alginate gelation relies on the presence of calcium ions. At 0 minutes, the gel may not be fully formed, affecting the availability and activity of the nanoparticles. After 10 minutes, the gel might have stabilized, facilitating the proper diffusion and activity of the CHN. 

The integration of CHN into IHC impression materials enhances cross-infection control and provides effective antibacterial and antifungal properties. This self-disinfecting approach eliminates the need for separate disinfection steps, improves patient safety, and saves clinical time. Additionally, CHN's antifungal efficacy is particularly beneficial in patients with compromised immunity. Because it is biocompatible and biodegradable, it supports eco-friendly dental practices. This study highlights CHN's superior antimicrobial action, its practical application within realistic time frames, and its innovation in enhancing infection control protocols without compromising material properties.

Limitations

The constraints of the research were that the mechanical and physical attributes of the IHC impression material in conjunction with 1% CHN were not investigated. Specimens were collected solely from the palatal region of the maxillary arch, whereas samples from the mandibular arch and other specific areas of the oral cavity were excluded. Furthermore, only 1% CHN was used in this investigation. Consequently, subsequent research should be undertaken utilizing a more substantial sample size with diverse concentrations of CHN incorporated into the IHC impression material while assessing both the antimicrobial and physicochemical properties.

## Conclusions

Chitosan, a multifaceted natural biomaterial, possesses a wide-ranging antibacterial spectrum and numerous advantageous characteristics. This investigation demonstrated that the incorporation of 1% CHN into the IHC impression material confers substantial antibacterial and antifungal properties. Consequently, this material can serve as a viable substitute for water in augmenting the antibacterial efficacy of IHC substances. This beneficial attribute can be harnessed for the self-disinfection of IHC impression materials. Additionally, the biocompatible and biodegradable nature of CHN aligns with sustainable practices in dentistry. Peak antimicrobial activity was observed at the 10-minute interval. Therefore, even pouring the impression at this time, cross-infection can be prevented due to the enhanced antimicrobial effect.

## References

[1] Oh WS, Park JM (2015). Use of irreversible hydrocolloid impression material to correct a defect in complete denture definitive impressions. J Prosthet Dent.

[2] Rubel BS (2007). Impression materials: a comparative review of impression materials most commonly used in restorative dentistry. Dent Clin North Am.

[3] Connor C (1991). Cross-contamination control in prosthodontic practice. Int J Prosthodont.

[4] McNeill MR, Coulter WA, Hussey DL (1992). Disinfection of irreversible hydrocolloid impressions: a comparative study. Int J Prosthodont.

[5] Suprono MS, Kattadiyil MT, Goodacre CJ, Winer MS (2012). Effect of disinfection on irreversible hydrocolloid and alternative impression materials and the resultant gypsum casts. J Prosthet Dent.

[6] Bendary IM, Omar AA, Goda RM, Ali AA, Lotfy KA, Shohayeb MM (2024). Evaluation of two different self-disinfection alginate impression material. BDJ Open.

[7] Jiang S, Chen FQ, Hu QQ (2024). Study on the effect of chlorogenic acid on the antimicrobial effect, physical properties and model accuracy of alginate impression materials. PeerJ.

[8] Elieh-Ali-Komi D, Hamblin MR (2016). Chitin and chitosan: production and application of versatile biomedical nanomaterials. Int J Adv Res (Indore).

[9] Hameed AZ, Raj SA, Kandasamy J, Baghdadi MA, Shahzad MA (2022). Chitosan: a sustainable material for multifarious applications. Polymers (Basel).

[10] Manikyamba YJ, Rama Raju AV, Suresh Sajjan MC, Bhupathi PA, Rao DB, Raju JV (2020). An evaluation of antimicrobial potential of irreversible hydrocolloid impression material incorporated with chitosan. J Indian Prosthodont Soc.

[11] (1987). Recommendations for prevention of HIV transmission in health-care settings. MMWR Suppl.

[12] Mushtaq MA, Khan MW (2018). An overview of dental impression disinfection techniques-a literature review. J Pak Dent Assoc.

[13] Amin WM, Al-Ali MH, Al Tarawneh SK, Taha ST, Saleh MW, Ereifij N (2009). The effects of disinfectants on dimensional accuracy and surface quality of impression materials and gypsum casts. J Clin Med Res.

[14] Singer L, Karacic S, Szekat C, Bierbaum G, Bourauel C (2023). Biological properties of experimental dental alginate modified for self-disinfection using green nanotechnology. Clin Oral Investig.

[15] Vanlalveni C, Lallianrawna S, Biswas A, Selvaraj M, Changmai B, Rokhum SL (2021). Green synthesis of silver nanoparticles using plant extracts and their antimicrobial activities: a review of recent literature. RSC Adv.

[16] Hardan L, Bourgi R, Cuevas-Suárez CE (2022). Disinfection procedures and their effect on the microorganism colonization of dental impression materials: a systematic review and meta-analysis of in vitro studies. Bioengineering (Basel).

[17] Bae K, Jun EJ, Lee SM, Paik DI, Kim JB (2006). Effect of water-soluble reduced chitosan on Streptococcus mutans, plaque regrowth and biofilm vitality. Clin Oral Investig.

[18] Kong M, Chen XG, Xing K, Park HJ (2010). Antimicrobial properties of chitosan and mode of action: a state of the art review. Int J Food Microbiol.

[19] Li S, Zhang H, Chen K (2022). Application of chitosan/alginate nanoparticle in oral drug delivery systems: prospects and challenges. Drug Deliv.

[20] Wathoni N, Herdiana Y, Suhandi C, Mohammed AF, El-Rayyes A, Narsa AC (2024). Chitosan/alginate-based nanoparticles for antibacterial agents delivery. Int J Nanomedicine.

[21] Yilmaz Atay H (2020). Antibacterial activity of chitosan-based systems. Functional Chitosan.

[22] N S SA, Pillai DS, Shanmugam R (2024). The antifungal activity of chitosan nanoparticle-incorporated probiotics against oral candidiasis. Cureus.

